# Natural killer cells during acute HIV-1 infection: clues for HIV-1 prevention and therapy

**DOI:** 10.1097/QAD.0000000000003319

**Published:** 2022-07-09

**Authors:** Aljawharah Alrubayyi, Sarah Rowland-Jones, Dimitra Peppa

**Affiliations:** a Nuffield Department of Clinical Medicine, University of Oxford, Oxford; b Division of Infection and Immunity, University College London; c Mortimer Market Centre, Department of HIV, CNWL NHS Trust, London, UK.

**Keywords:** acute HIV-1 infection, antiretroviral therapy, cytomegalovirus, HIV-1, natural killer cells, therapeutic treatment

## Abstract

Despite progress in preexposure prophylaxis, the number of newly diagnosed cases with HIV-1 remains high, highlighting the urgent need for preventive and therapeutic strategies to reduce HIV-1 acquisition and limit disease progression. Early immunological events, occurring during acute infection, are key determinants of the outcome and course of disease. Understanding early immune responses occurring before viral set-point is established, is critical to identify potential targets for prophylactic and therapeutic approaches. Natural killer (NK) cells represent a key cellular component of innate immunity and contribute to the early host defence against HIV-1 infection, modulating the pathogenesis of acute HIV-1 infection (AHI). Emerging studies have identified tools for harnessing NK cell responses and expanding specialized NK subpopulations with adaptive/memory features, paving the way for development of novel HIV-1 therapeutics. This review highlights the knowns and unknowns regarding the role of NK cell subsets in the containment of acute HIV-1 infection, and summarizes recent advances in selectively augmenting NK cell functions through prophylactic and therapeutic interventions.

## Introduction

An estimated 1.5 million acquired HIV-1 in 2020 around the world, with more than 30 million people worldwide currently living with HIV-1 [1]. There is, therefore, an urgent public health need to develop new preventive and curative strategies to tackle the global HIV-1 epidemic. A better understanding of the early immunological events during acute HIV-1 infection (AHI) could provide vital clues for the design of vaccine development, treatment and novel cure interventions.

AHI refers to the first months following HIV-1 exposure and is associated with exponential viral replication and the establishment of a stable viral load set-point [2]. During AHI, the recognition of viral pathogen-associated molecular patterns (PAMPs) initiates a signalling cascade that triggers innate intracellular antiviral responses aimed at controlling viral replication [3]. This innate cell-intrinsic response involves secretion of multiple factors including cytokines and chemokines, which activate innate immune cells and attract them to the site of infection [3]. Emerging evidence suggests that innate immune responses are key contributors to the containment of the viral replication during AHI and an important determinant of the level of immune activation and subsequent disease outcome [4].

NK cells constitute a critical component of the innate lymphocyte population, which occupy a unique niche in the immune system, bridging innate and adaptive immune responses [5]. Their fundamental role in antiviral defence has been unveiled by the increased susceptibility of patients with congenital NK cell immunodeficiencies to viral infections, particularly with herpesviruses [6,7] and viral evasion strategies to elude NK cell-mediated-control [8].

**Fig. 1:**
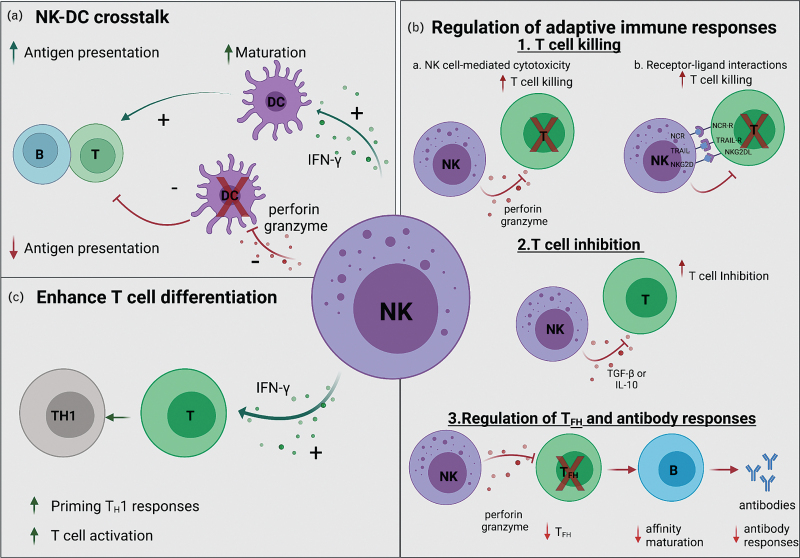
Natural killer cell immunoregulatory functions.

Human NK cells have been traditionally classified based on the expression of the neural cell adhesion molecule CD56 into CD56^dim^ and CD56^bright^ NK cells [9,10]. CD56^bright^ NK cells are cytokine-producing cells whereas CD56^dim^ NK cells are known for their effective cytotoxic ability [11,12]. NK cells exert cytotoxicity via exocytosis of cytolytic granules or perforin-mediated mechanisms or via pathways involving the engagement of extracellular death receptors (e.g. Fas, TRAIL-Rs) on target cells [13,14], or indirectly through Fc-mediated effector responses, known as antibody-dependent cellular cytotoxicity (ADCC) [5]. In addition to cytotoxicity, NK cells are potent producers of chemokines and cytokines, including tumor necrosis factor (TNF)-α and interferon (IFN)-γ [15]. Furthermore, they interact with other immune subsets, including dendritic cells, CD4^+^ and CD8^+^ T cells, shaping antiviral responses [16–18]. Interaction between NK cells and dendritic cells can result in activation of both subsets, upregulating NK cell functionality and inducing maturation of dendritic cells, leading to efficient virus-specific adaptive immune responses [19,20]. However in HIV-1 infection, impairement of both NK cell and dendritic cell functions can lead to dysregulated crosstalk, with potentially important consequences for the development of antiviral B-cell and T-cell responses [21]. Such interactions during AHI remain, however, poorly described. NK cells can enhance or suppress adaptive B-cell and T-cell responses influencing the outcome of infection (reviewed in [17,22,23]) (Fig. 1). In human viral infections, we have previously shown how NK cells can limit HBV-specific T cells and modulate the induction of broadly neutralizing antibodies in HIV-1 infection [14,24]. Whether this immunoregulatory capacity of NK cells during the early stages of HIV-1 infection is protective or detrimental to the host remains currently unclear.

(a) NK cells can directly interact with, and positively or negatively modulate dendritic cells (DCs). Crosstalk between NK cells and dendritic cells augments dendritic cell maturation and enhances their antigen-presenting capacity, which in turn promotes adaptive immune rersponses. (b) NK cells can also interact directly or indirectly with CD4^+^ and CD8^+^ T cells and modulate the magnitude of T-cell and B-cell responses through different mechanisms depicted. (c) NK cell-derived cytokines, such as IFN-γ, promotes T-cell differentiation and enhances T-cell responses. IFN-γ, interferon-gamma; NCRs, natural cytotoxicity receptors; TRAIL, tumor necrosis factor (TNF)-related apoptosis-inducing ligand.

The activation and function of NK cells is regulated by the balance of signals from an array of germline-encoded inhibitory and activating receptors [25]. NK cell activating receptors, including natural killer group 2 member D (NKG2D), the natural cytotoxicity receptors (NCRs), NKp30, NKp44 and NKp46, are involved in the recognition of molecules upregulated by stress and virus infections [26]. The inhibitory receptors, including CD94-NKG2A or inhibitory killer cell immunoglobulin-like receptors (iKIRs), are involved in NK cell inhibition and maintenance of self-tolerance through interaction with nonclassical and classical major histocompatibility complex (MHC) molecules [27]. The interaction between self-MHC class I molecules and iKIRS or nonclassical MHC [human leucocyte antigen E (HLA-E) in humans] and CD94/NKG2A are critical not only for evaluating the absence of ‘self’ but also for the education of NK cells; a process required for NK cells to acquire effector responses or self-tolerance through their development (reviewed in [28,29]). The activity of NK cells is further tuned by local environmental factors, which can modulate their survival and function [30].

Accumulating evidence supports an important role for NK cells in the control of HIV-1 viremia, including long-term HIV-1 suppression in the VISCONTI cohort of posttreatment controllers [5]. As a component of the early response to virus infection, NK cells have been associated with lower risk of HIV-1 acquisition [31]. Both epidemiological and mechanistic studies have linked NK cell activity and HLA-mediated immune control of HIV-1. Certain HLA-Bw4 alleles in combination with KIR3DL1 and KIR3DS1 have been associated with slower disease progression [32,33]. Additional studies have demonstrated the protective effects of KIR3DL1/S1/HLA-Bw4 combinations in HIV-1 infection [34,35], whereas specific KIR2DL2/3 genotypes have been associated with HIV-1 sequence mutations, suggesting NK cell-mediated immune pressure [36]. More recently HLA-B haplotypes that promote NK cell education via NKG2A, were shown to exacerbate the negative effect of high HLA-A expression on HIV-1 control through enhanced expression of HLA-E and increased NKG2A-mediated inhibition of NK cells, leading to decreased killing of HIV-1 infected target cells [37]. In addition to HIV-1 infection, enhanced NK cell activation and effector functions were also detected in acute hepatitis B virus (HBV) infection [38–40]. In acute hepatitis C virus (HCV) infection, the early interaction between NK cell receptor KIR2DL3 and HLA-C1 was associated with enhanced viral clearance [41,42], whereas the combination of KIR2DL3 and KIR2DS3 with HLA-C2 was linked to increased viral pathogenesis [43]. Collectively, these data suggest that NK cells are a significant component of the early antiviral response against different human viral infections that can influence disease outcome, depending on host genetics and degree of functional responsiveness.

This review summarizes recent advances in our understanding of early NK cell responses during AHI that could pave the way towards the development of new or complementary NK cell-based prophylactic strategies.

## Natural killer cell-mediated control of HIV-1 replication during acute infection and progressive dysregulation

**Fig. 2:**
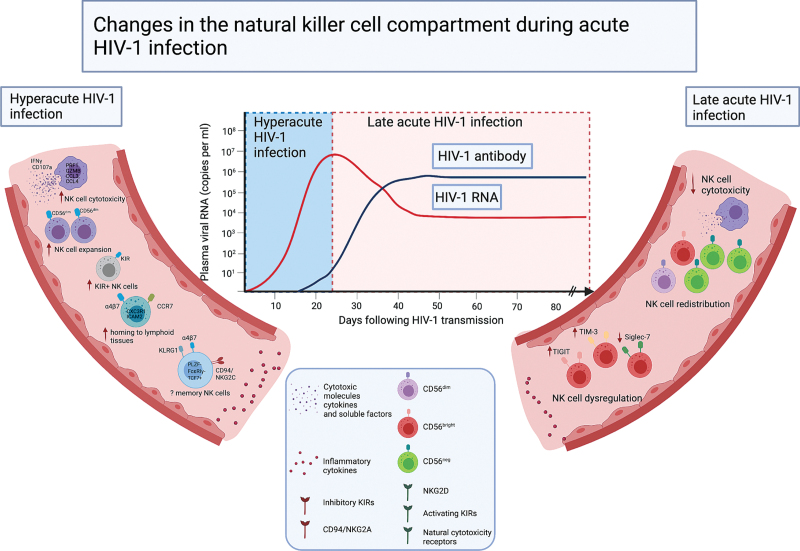
Changes in the natural killer cell compartment during acute HIV-1 infection.

The functional and phenotypic characteristics of the HIV-specific cytotoxic T-cell responses in AHI that correlate with low viral set-point and enhanced clinical outcomes are well studied [44,45]. However, much less attention has been given to innate cellular responses during AHI and how these might contribute to the viral control and the development of adaptive responses. Previous work has demonstrated that, following viral transmission, the absolute number of NK cells increases during hyperacute infection [i.e. Fiebig stages I and II; prior to development of detectable HIV-1 antibodies [46,47]], preceding adaptive responses (Fig. 2) [48]. This expansion of NK cells at the earliest window of acute HIV-1 infection is dominated by an increase in CD56^dim^, cytotoxic NK cells and a depletion of CD56^bright^ NK cells [49]. Increased NK cell activity on the basis of induction of IFN-γ and CD107a against MHC class I devoid target cells has been detected during the hyperacute phase [48,49]. The levels of NK cell degranulation associated with HIV-1 replication and were found to correlate inversely with the magnitude of HIV-specific CD8^+^ T cells, in keeping with distinct trajectories of NK cell and T-cell effector functions during the very early phases of HIV-1 infection [48,50]. More recently, the dynamics of immune cell responses following HIV-1 exposure and during the first month of infection were described in the FRESH cohort of young women in South Africa [51]. The level of genes associated with NK cell cytotoxicity (*PRF1, GZMB*) and chemokine signalling pathway (*CCL3, CCL4*) were upregulated before or during peak viremia [51]. Although these two pathways were linked during the earliest stage of infection, the strength of the correlation declined over time, suggesting maximal NK cell plasticity near the time of peak viremia [51]. Notably, the presence of proliferative and cytotoxic NK cells at the earliest stage, 1 week following HIV-1 infection, correlated with long-term viral control [51]. Along these lines, robust NK cell functionality during the first 3 months of the infection has been linked to a higher CD4^+^ T-cell count (>500 cells**/μl**) for over 2 years in the absence of antiretroviral treatment, suggesting a beneficial role for NK cells in controlling disease progression [52]. An early NK cell-mediated ADCC response has also been associated with a lower viral set point [53], a key predictor of HIV-1 disease outcome [54,55], and a higher CD4^+^ T-cell count [56]. Overall, these findings suggest that NK cells are a key cytotoxic effector population at the initial phase of infection with a potentially important role in the control of peak viremia.

Recent studies of the NK cell compartment during AHI have revealed different NK cells dynamics during the first months following infection, which can be divided into two stages; before seroconversion (hyper-acute HIV-1 infection) and after seroconversion (late acute HIV-1 infection) based on the detection of HIV-1-specific antibodies in clinical diagnostic assays. During hyperacute HIV-1 infection, circulating NK cells are expanded with an increase in their antiviral responses and trafficking markers, followed by a moderate decline in the total NK cell number and pathological redistribution of NK cell subsets in late acute HIV-1 (AHI) infection. ADCC, antibody-dependent cellular cytotoxicity; HLA-I, HLA class I molecules; KIRs, killer cell immunoglobulin-like receptors; NKG2C, natural killer cell activating receptor; PLZF, the transcription factor promyelocytic leukaemia zinc finger protein. FcγRIIIa:CD16 receptor binds to the Fc portion of the antibodies; FcεRγ, signaling protein.

However, the early expansion of NK cells is not maintained during the later stages of acute HIV-1 infection (i.e. Fiebig stages III, IV and V; approximately between 1 and 6 months post transmission [46]), suggesting that this could be a transient phenomenon [50,52]. This is in keeping with a more pronounced upregulation in gene expression associated with NK cell proliferation during the first week of infection [51], whilst no significant changes in the frequency of dividing NK cells have been detected at the later stages of acute infection [50].

The dynamics of NK cell responses during AHI, including NK cell compartmentalization and trafficking, remain incompletely understood. Limited information on phenotypic characterization from initial studies showed an expansion of KIR^+^ NK cells [57], although specific KIRs were not distinguished in this study [50]. Further work demonstrated an increased frequency and polyfunctionality of KIR2DL1^+^ and KIR2DL2/3^+^ NK cells in primary HIV infection in participants encoding for their cognate HLA-C haplotype. This study highlights how the interaction between KIR2DL1^+^ and KIR2DL2/3^+^ NK cells and their respective HLA-C ligands can lead to educated and more functionally potent NK cells during AHI [57].

Higher expression of CCR7 on NK cells, a receptor for homing to lymphoid tissues, has been described during the early phase of infection but not during the later stages of infection [50]. More recently, genes associated with NK cell trafficking (*CXC3R1* and *ICAM2*) were shown to be upregulated during the first week of infection and to persist throughout the first month of AHI [51], suggesting that NK cell homing to lymphoid tissues occurs very early in infection. Additional studies are required to determine how changes in the NK cell trafficking *in vivo* influence the viral load set point in the acute phase of HIV-1 infection. It is plausible that NK cells via virtue of their ability to target T follicular helper (T_FH_) cells [24], a significant contributor to the HIV-1 reservoir, may influence the size of the viral reservoir that is established after the acute phase of infection. This is supported by studies in simian immunodeficiency virus (SIV) infection, where NK cells have been shown to play a key role in controlling nonpathogenic SIV infection by homing into lymph node follicles, a likely viral reservoir site [58]. More recent data in humans demonstrated that CXCR5^+^ NK cells accumulate in lymph nodes and correlate negatively with HIV-1 DNA levels, suggesting that these NK cell populations may be a promising target for functional cure strategies [59].

Currently, there is limited information on the role of NK cells in mucosal tissues, a portal of viral entry during AHI. In a pathogenic vaginal SIV challenge model, NK cell recruitment to the female genital tract (FGT) was detected following exposure to SIVmac251 and during the first week of vaginal inoculation [60]. Although, mucosal NK cells secreted cytokines and chemokines, including IFN-γ and CCL3, these cells lacked markers of activation (CD38, HLA-DR and CD69), proliferation (Ki67) and cytotoxicity (CD107a), suggesting an impairment of their conventional cytotoxic role [60]. Interestingly, vaccination with SIVmac239Δnef, which has been shown to protect against high-dose vaginal challenge in animals [61–63], was not associated with increased NK cell recruitment to the genital mucosa, suggesting a limited involvement of NK cells in vaccine-induced protection [60]. Further work is required to fully unravel the role of NK cells at mucosal surfaces and to evaluate whether NK cell responses measured in the systemic circulation [50] are present at the sites of HIV-1 exposure and the potential mechanism of protection.

Findings from early studies indicate a progressive dysregulation of NK cell functional responses during primary HIV-1 infection, not solely attributed to the emergence of anergic CD56^−^CD16^+^ NK cells, suggesting wider defects of NK cell activation with progressive infection [49,50,64]. Continuous activation and inflammation during acute HIV-1 infection has been shown to result in altered expression of the immunoregulatory molecule T-cell immunoglobulin and mucin-domain containing-3 (Tim-3) [65] and appearance of TIGIT^+^NK cells with decreased NK cell functionality [66]. Siglec-7, an inhibitory receptor highly expressed by NK cell subsets [67], has also been proposed as an early marker for NK cell dysregulation in HIV-1 infection [68]. NK cell subsets with a decreased expression of Siglec-7 were expanded during AHI and were characterized by impairment of NK cell degranulation and cytokine production [68]. The expansion of Siglec-7^−^ NK cells in early infection is followed by NK cell subset redistribution and the emergence of dysfunctional CD56^−^CD16^+^ NK cells in chronic infection [68].

Our understanding of the full extent of interactions between NK cells and HIV-1-infected target cells leading to NK cell activation/expansion or exhaustion remains currently incomplete. In view of the current standard of care (i.e. treatment at detection) and limited sample availability, nonhuman primate (NHP) models could provide valuable opportunities to assess the role of NK cell subsets in mediating HIV-1 control through direct or indirect (i.e. regulation of dendritic cells or adaptive responses) functions, especially within key tissue compartments.

## Early antiretroviral therapy initiation during acute HIV-1 infection and its impact on the natural killer cell compartment

Although antiretroviral therapy (ART) introduction mitigates some defects in the NK cell compartment, altered NK cell subset re-distribution and impaired functionality persist despite successful virological suppression [69–72]. Data from NHP early treatment models and limited evidence from ART initiation studies during acute infection suggest that the trajectory of HIV-related inflammation and the degree of functional restoration may vary with the timing of ART initiation [73–75].

Profiling of circulating NK cells in individuals treated during AHI has shown that early ART administration partially restricts NK cell redistribution by moderating CD56^bright^ NK cell depletion [49,75,76]. An in-depth longitudinal study of immediate ART initiation in a cohort of neonates with HIV-1 infection who initiated ART shortly after birth (EIT), compared with infants treated during the first year, showed that EIT infants display a distinct NK cell profile [77]. This is characterized by increased frequency of CD56^dim^CD16^−^ subsets and reduced proportion of the functionally impaired CD56^−^CD16^dim^ subset after 2 years of ART treatment [77]. EITs also exhibited differential dynamics of NK cell subpopulations reflected in the expression of NKG2D, NKp30 and CD161 [77]. NKp30 expressing CD56^dim^ NK cells were found to negatively correlate with intact proviral sequences, whereas CD56^−^CD16^+^ NK cells expressing NKG2D and CD161 were positively associated with intact proviral sequences in EIT neonates [77]. Although these data are limited by the sample size, they suggest a role for NK cell responses in shaping the viral reservoir in neonates [77]. This notion is further supported by another study in adults in whom a correlation between frequency of circulating CD56^−^CD16^bright^ and CD56^dim^ NK cells and the level of HIV-1 DNA in rectal CD4^+^ T cells has been demonstrated [75]. Notably rectal NK cell subsets differed depending on whether ART was initiated during acute or chronic infection [75]. Lower CD56^bright^ NK cell frequencies, the major cytokine producers, were observed in the group that started ART in acute infection, which could influence the local pro-inflammatory environment reducing HIV-1 replication and preserving CD4^+^ T cells [75]. Further work is required to distinguish subpopulations of NK cells responsive to early ART treatment and how these subsets contribute to the overall reconstitution of adaptive immune responses and size of viral reservoirs within key effector sites.

## Adaptive/memory natural killer cell responses in acute HIV-1

**Fig. 3:**
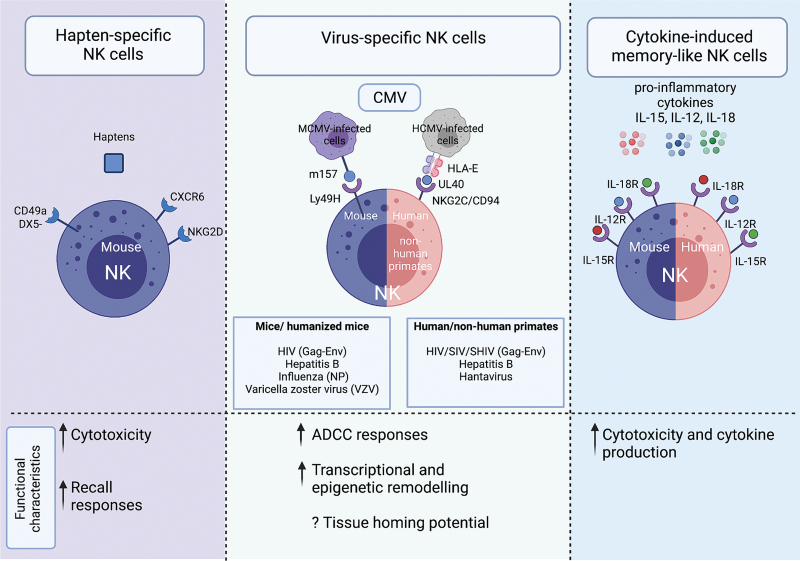
Summary of different adaptive/memory natural killer cell subsets described in humans and animal models.

It is increasingly recognized that distinct NK cell subsets can expand and form a long-lasting pool of lymphocytes with adaptive/memory traits. Exposure to distinct inflammatory cytokines, chemical haptens, vaccine antigens, or cytomegalovirus (CMV)-derived peptides results in preferential expansion of a specialized NK cell subpopulation with enhanced responsiveness and ADCC capacity (Fig. 3) [78–82]. In humans, the best characterized adaptive NK cell subset arises in response to CMV infection [81,82], an almost universal co-infection in HIV-1 cohorts. These adaptive NK cell subpopulations are characterized by higher expression of NKG2C, the activating counterpart of NKG2A, and have a skewed receptor repertoire towards differentianted CD57^+^ NK cells with a preferential oligoclonal pattern of KIRs for self HLA-C [78,80,81]. They are also delineated by downregulation of key adaptor molecules, such as FcεRγ and EAT-2, and the transcription factor promyelocytic leukaemia zinc finger (PLZF) [83–85]. We and others have described an adaptive reconfiguration of NK cells in HIV-1 infection, partially driven by CMV co-infection, becoming apparent during the early stages of HIV-1 infection and more pronounced during chronic infection [86,87]. Such populations display retained responses to CD16 stimulation [88,87] and superior ADCC responses to overlapping HIV-1 peptides [84], with implications for developing strategies exploiting their Fc-dependent functions. It is also conceivable that the downregulation of HLA-C by most primary HIV-1 clones may also facilitate ‘missing-self’ recognition of HIV-1-infected cells by CMV-primed adaptive NK cells.

(a) NK cell immunological memory has been described to haptens in mice, in a subset of NK cells residing in the liver. (b) Antigen-specific NK cell responses described in mice and humans/nonhuman primates. During MCMV infection, the viral m157 protein is recognized by Ly49H-expressing NK cells, which leads to expansion of CMV-specific NK cells. In human, NK cells expressing NKG2C/CD94 recognize HLA-E bound CMV peptides (UL40) resulting in virus specifc NK cell responses. Virus-specific NK cell responses have also been identified in other viral infections, including HIV-1 and SIV, in humanized mice, nonhuman primates and humans. (c) Cytokine-induced memory-like NK cells in mice and humans can be generated by stimulation with IL-12/15/18. CMV, cytomegalovirus; NK, natural killer; SIV, simian immunodeficiency virus.

Limited evidence from human studies demonstrated that the presence of NK cells with an adaptive-like phenotype (CD57^+^NKG2C^+^), during early HIV-1 infection, is inversely correlated with HIV-1 viral load and associated with a lower viral set point [89,90]. Such data suggest that NK cells with adaptive features, despite the limited profile used for their identification, may constitute a readily armed population that confers better HIV-1 control, highlighting their potential value in early infection as a prognostic marker for monitoring HIV-1-infected patients and potentially remission.

In addition to CMV-driven adaptive NK cells, durable antigen-specific NK cell responses have been reported in primates after SIV infection and vaccination [80]. More recently mechanistic evidence of human NK cell HIV-specific memory was reported and suggested as a potential correlate of HIV-1 control in a subgroup of HIV-1-infected individuals, elite controllers, who maintain viral load suppression in the absence of ART [91]. HIV-specific responses were dependent on the interaction between the activating NK cell, NKG2C receptor and its ligand HLA-E bound to HIV-1 Gag or Env peptides [91]. Comprehensive transcriptomic and phenotypic analysis of these antigen-specific NK cells showed, in addition to expression of NKG2C, elevated expression of KLRG1, which has also been reported to define NK cell subsets that mediate HBV-specific responses [92] and higher α4β7 expression, indicating mucosal homing potential [91,93,94]. Terminally differentiated NKG2A^low^ CD16^+^ NK cells with an adaptive gene expression profile (low expression of *ZAP70*, *FcεRγ* and *SYK* and high expression of *GRAP2*), and HLA-E-restricted cytotoxicity against SIV-infected cells have also been associated with strong viral control in the secondary lymphoid tissues of African green monkeys [95].

Antigen-specific memory could, however, develop independent of the NKG2C/HLA-E axis and other pathways may be complementary or alternative to this pathway. The inflammatory milieu/higher levels of LPS in HIV-1 infection could further drive the expansion of NK cell subsets with cell intrinsic memory properties [96,97]. Recently the presence of a memory-like NK cell population, delineated by CD94^+^CD56^hi^ and high expression of the transcription factor TCF7, was reported, displaying higher cytotoxicity against HIV-infected CD4^+^ T cells and correlating with HIV-1-induced inflammation [97]. Interestingly prior studies have suggested differences in HIV-1 pathogenesis and acute retroviral syndrome (ARS) prevalence depending on subtype and the presence of soluble inflammatory markers [98,99]. However, differences in innate immune responses between different HIV-1 subtypes remain under-investigated. A stronger innate signature and high levels of IP-10 during hyperacute AHI have been associated with ARS [99]. IP-10 is increased in many acute viral infections and correlates with expansions of adaptive NK cells in HIV-1-infected individuals [84]. It is, therefore, plausible that the inflammatory environment induced by infection with different HIV-1 subtypes drives the differential expansion of preexisting populations of NK cells with memory and specific lymph node/mucosal homing features that contributes to HIV-1 control and influences the development of adaptive responses. A better understanding of the mechanisms implicated in the generation of adaptive/memory NK cells is critical for developing NK cell-based immunotherapies.

## Harnessing natural killer cell functions for preventive and therapeutic use with emphasis on vaccination and viral reservoir elimination

Emerging evidence of the potent and diverse functions of NK cells during the initial stages of HIV-1 infection and the recent advances in understanding their adaptive/memory properties hold great promise for exploiting their potential in HIV-1 immunotherapy. HIV-1 prophylactic and therapeutic strategies can target NK cell activation and/or enhance HIV-specific NK cell responses to augment viral control and eradication of viral reservoirs (Table 1).

**Table 1:** Selected preventive and therapeutic approaches targeting natural killer cell activity and associated impact on HIV-1 viremia.

		Type	Host	Mode of action	Impact on viremia	Reference
Enhancing NK cell activation and ADCC activity	Toll-like receptor (TLR) agonists	TLR7	Rhesus monkeys	↑ NK cell activation	↓ The size of the SIV latent reservoir and delayed viral rebound following ART discontinuation	[100,107]
	Broadly neutralizing antibodies (bNAbs)	bNAbs PGT121 + TLR7	HIV-1 infected targets Rhesus monkeys	ADCC ↑ NK cell activation	Delayed viral rebound following ART interruption	[106,107]
	NK cell immunostimulatory cytokines	Soluble IL-15 + vorinostat	Human	↑ NK cell activation	↑ Clearance of latently HIV-infected cells after reactivation	[112,116]
		Soluble IL-15; IL-15 superagonists	Humanized mice	↑ NK cell activation	↑ Anti-HIV activity and suppress acute HIV-1 infection	[111]
		IL-15 pre-treatment of NK cells	Human	↑ NK cell activation	↑ Killing of HIV-1 infected cells by vaccine induced antibodies mediating ADCC	[112]
		Pegylated IFN-α treatment	HIV-1/ hepatitis C virus-coinfected patients	↑ NK cell cytotoxicity	↓ Cell-associated proviral DNA	[115]
	Latency reversing agents	Histone deacetylase inhibitors (HDACi) panobinostat and SUW133	Human	↑ Susceptibility to NK cell mediated killing	↓ HIV-1 DNA levels and delayed viral rebound	[118–120]
Enhancing NK cell recruitment into key sites and NK cell engineering	NK cell recruitment to HIV-1 viral reservoir sites	Injection of mature DCs and R848 and Ribi	Mice	↑ NK cell recruitment to lymph nodes	↑ TH1 polarization	[117]
	NK cell engineering	CAR-modified HSPCs	Humanized mice	Differentiate into functional T and NK cells	↓ HIV replication	[101]
		Universal CAR-NK cells	Human	Recognize different epitopes of gp160	↑ Killing of HIV-1 infected cells	[102]
Eliciting memory NK cell responses		Vaccination	Macaques	↑ Antigen-specific responses against HIV-1	↑ Killing Gag- and Env-pulsed target cells	[80]
		Adoptive transfer of virus sensitized NK cells	Humanized mice	↑ Memory responses against HIV-1	↑ Animals’ survival following challenge with virus-like particles (VLPs) containing HIV-1 derived gag/env	[127]

NK, natural killer.

### Enhancing natural killer cell activation and antibody-dependent cellular cytotoxicity activity

Administration of antibodies that are capable of mediating strong ADCC responses is a promising strategy to enhance clearance of infected cells through engagement of Fc-receptors on NK cells [103]. In particular the generation of anti-HIV-1 broadly neutralising antibodies (bNAbs) with greater breadth and efficacy with the capacity to suppress viral replication and potential for ADCC, is of great interest in current HIV-1 functional cure approaches [104–106]. Combination of bNAb administration with latency reversal agents and NK cell-stimulating agents may be an effective approach for viral reservoir clearance. Administration of bNAb (PGT121) together with the Toll-like receptor 7 (TLR7) agonist resulted in NK cell activation and a delayed viral rebound following ART interruption in (SHIV)-SF162P3-infected rhesus monkeys [107]. A further study showed that combining the latency reversing agent SUW133 with the protein kinase C modulator and allogeneic human peripheral blood NK cells, during ART treatment interruption, eliminated the viral reservoir in a subset of humanised mice [108].

Myeloid and T-cell-derived cytokines (IL-12, IL-15, IL-18 and IL-2) have been well documented to contribute to NK cell priming and activation [109,110]. Stimulation of NK cells with a recombinant IL-15 super-agonist *in vitro* and *in vivo* augmented their cytotoxic effector responses, leading to NK-cell-mediated killing of HIV-infected T cells and suppression of acute HIV-1 infection in a humanized mouse model [111]. Similarly, IL-15 pretreated NK cells exhibited a higher ADCC activity mediated by vaccine-induced antibodies, in response to HIV-infected cells in the HVTN-100 vaccine trial [112]. Tissue-resident NK cells in the lymph node, mainly CD56^bright^ subsets, which display low cytolytic ability, can upregulate cytotoxicity by exposure to low amount of IL-2 [113,114]. Similarly pegylated Interferon-α (PEG-IFN-α)-treated HCV/HIV-1 co-infected individuals showed a reduction in cell-associated proviral DNA associated with PEG-IFN-α-induced CD56^bright^ NK cell activation [115]. IL-15 and/or other cytokines could, therefore, be important adjuvants in vaccine regimens [112], as well as an important element in strategies aimed at eliminating the viral reservoir [116].

### Enhancing natural killer cell recruitment into key sites and natural killer cell engineering

Different approaches can be considered to aid NK cell recruitment into key sites for viral reservoir elimination, such as lymph nodes, in combination with latency reversal agents. In animal models, recruitment of peripheral CD56^dim^ NK cells to the lymph nodes can be induced by injection of mature dendritic cells [117] and certain adjuvants, including R848 and Ribi [117]. Histone deacetylase inhibitors (HDACi) employed for HIV-1 reservoir reactivation can induce alterations in the expression of NK cell receptor ligands on the infected CD4^+^ T cells, leading to improved NK cell-mediated killing of HIV-1-infected T cells [118–120]. ART-treated HIV-1-infected individuals treated with the HDACi, Panobinostat, displayed 70–80% reduction of HIV-1 DNA viremia and delayed viral rebound following treatment interruption, which related to NK cell activity [118]. These data highlight the potentially important role of NK cells in modulating the effects of latency reversal agents on the viral reservoir.

Chimeric antigen receptor (CAR)-engineered NK cells are also emerging as a promising new tool with therapeutic potential. Recently, CAR-expressing human NK cells were engineered to selectively eliminate germinal centre (GC) PD-1^high^ T_FH_ subsets [121]. T_FH_ T cells are highly permissive to HIV-1 infection and a major source of HIV-1 reservoirs [122]. Engineered expression of CXCR5 on CAR NK cells could enhance NK cell recruitment to the sites of viral reservoirs. This would mirror trafficking pattern of CXCR5^+^ NK cells observed in SIV-infected AGM and HIV-1-infected individuals [58,59]. Hence this could represent a novel strategy to improve clearance of latently infected T cells in key site of viral reservoirs [123].

### Inducing memory natural killer cells for preventive stategies

Vaccination strategies aimed at inducing memory NK cell subpopulations in combination with induction of classical memory responses (virus-specific T cells and/or bNAbs) is an exciting area that can circumvent some of the unwanted immunoregulatory effects of conventional NK cells [17,24,87]. NK cells isolated from humanized mice vaccinated with HIV-encoded envelope protein displayed vaccine-dependent, antigen-specific memory responses [82]. Splenic and hepatic NK cells from Ad26-vaccinated macaques specifically lysed antigen-matched targets and these responses could be detected 5 years after Ad26 vaccination [80]. The specificity of adaptive NK cells to HLA-E presented peptides [73], including the previously unappreciated ability of HLA-E to bind HIV-1 peptides [91], leading to activation of virus-specific NK cells opens up the possibility for further refining these approaches. The significance of HLA-E-restricted responses has received significant attention in the HIV-1 vaccine field. This is following observations of broad HLA-E-restricted CD8^+^ T cells as important correlates of immune protection in vaccinated macaques with a CMV-vectored SIV vaccine [124–126]. Such strategies that encourage HLA-E-dependent responses could lead to the induction of HIV-1-specific memory NK cells with the ability to react to a broader range of antigens from the same or different viruses, complementing T-cell responses.

## Concluding remarks and remaining knowledge gaps

Our knowledge of immune responses during early infection has been hampered by the fact that identification of infected individuals within the first weeks of infection is challenging [2]. This leads to many unanswered questions about the immune dynamics during AHI, particularly innate immunity, which has been much less investigated in AHI compared with adaptive immune responses. Further research is required to identify the NK cell subpopulations that contribute to elimination of the transmitted virus especially within tissues, crosstalk with other immune cells and the basic mechanisms underlying NK cell memory. Nonetheless, insights from studies in AHI highlight the role of NK cells in controlling viremia at the early stages of infection. By virtue of their effector functions, they can eliminate HIV-1-infected cells as well as secrete cytokines and chemokines that can influence antiviral responses and limit HIV-1 infectivity of target cells. NK cells homing into lymphoid follicles/mucosal tissues may also influence the size of the viral reservoir, lending support to combined strategies to enhance NK cell function and directing them at key sites. Another favourable property of NK cells is their capacity for ADCC, which appears to be a critical component of the anti-HIV-1 immune response and has been linked to phenotypes of viral control. Several approaches are, therefore, aimed at triggering NK cell-mediated ADCC. An exciting development in the field of NK cell biology is the description of NK cells with adaptive/memory properties. Their selectivity/specificity and amplified functional responses make them highly desirable targets for clinical exploitation and vaccine design. Increasing our understanding of the early imprinting effects of HIV-1 infection on the NK cell compartment and determining the contribution of specific NK cell subsets in the recognition and clearance of HIV-1-infected cells will be critical in informing new and well tolerated therapeutic approaches as an alternative or combination strategy for a functional cure.

## Acknowledgements

We would like to acknowledge and thank Ester Gea-Mallorqui and Natasha Fisher-Pearson for their assistance in preparing this manuscript.

The illustration figures were created with BioRender.

Funding: this work was supported by Saudi Ministry of Education graduate student grant (A.A.), BHIVA and NIH R01AI55182 (D.P.).

Author contributions: A.A., S.R.J. and D.P. contributed to writing and editing the manuscript. All authors listed have participated in a direct and intellectual contribution to the work, and approved it for publication.

### Conflicts of interest

There are no conflicts of interest.
